# Restoration age affects microbial‐herbaceous plant interactions in an oak woodland

**DOI:** 10.1002/ece3.11360

**Published:** 2024-05-02

**Authors:** Rachel A. Brant, Christine E. Edwards, John Leighton Reid, Burgund Bassüner, Brad Delfeld, Noah Dell, Scott A. Mangan, Victoria de la Paz Bernasconi Torres, Matthew A. Albrecht

**Affiliations:** ^1^ Missouri Botanical Garden St. Louis Missouri USA; ^2^ Department of Biological Sciences Arkansas State University Jonesboro Arkansas USA; ^3^ Present address: School of Plant and Environmental Sciences Virginia Tech Blacksburg Virginia USA

**Keywords:** conservative species, DNA metabarcoding, oak woodland, soil microbial communities

## Abstract

In degraded ecosystems, soil microbial communities (SMCs) may influence the outcomes of ecological restoration. Restoration practices can affect SMCs, though it is unclear how variation in the onset of restoration activities in woodlands affects SMCs, how those SMCs influence the performance of hard‐to‐establish woodland forbs, and how different woodland forbs shape SMCs. In this study, we quantified soil properties and species abundances in an oak woodland restoration chronosequence (young, intermediate, and old restorations). We measured the growth of three woodland forb species when inoculated with live whole‐soil from young, intermediate, or old restorations. We used DNA metabarcoding to characterize SMCs of each inoculum treatment and the soil after conditioning by each plant species. Our goals were to (1) understand how time since the onset of restoration affected soil abiotic properties, plant communities, and SMCs in a restoration chronosequence, (2) test growth responses of three forb species to whole‐soil inoculum from restoration sites, and (3) characterize changes in SMCs before and after conditioning by each forb species. Younger restored woodlands had greater fire‐sensitive tree species and lower concentrations of soil phosphorous than intermediate or older restored woodlands. Bacterial and fungal soil communities varied significantly among sites. Forbs exhibited the greatest growth in soil from the young restoration. Each forb species developed a unique soil microbial community. Our results highlight how restoration practices affect SMCs, which can in turn affect the growth of hard‐to‐establish forb species. Our results also highlight that the choice of forb species can alter SMCs, which could have long‐term potential consequences for restoration success.

## Graphical abstract

In this study, we determined how soil microbial communities vary across an oak restoration chronosequence. We measured the growth of three woodland forb species when inoculated with live whole‐soil from young, intermediate, or old oak restorations. Using DNA metabarcoding, we characterized soil microbial communities of each restoration soil as well as soil after conditioning by each plant species. We find that plants grew better in young restored soils, and each plant species cultivated a unique soil microbial community.

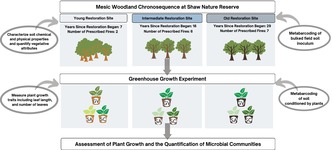

## 1 INTRODUCTION

Human activities have degraded natural ecosystems globally, resulting in declines in biodiversity and ecosystem functioning (IPBES, 2019). In response to these declines, ecological restoration is an increasingly important strategy to recover biodiversity, ecosystem function, and historical disturbance regimes in degraded sites (Strassburg et al., 2020). Traditionally, restoration has focused on re‐establishing plant communities and aboveground ecological processes, often overlooking the taxa and processes occurring belowground (Ezeokoli et al., 2020; Sun et al., 2017; Van Der Heyde et al., 2020). However, recent studies indicate soil microbiota may mediate plant establishment and shape the long‐term trajectories of restored communities (Coban et al., 2023; Wubs et al., 2016). Soil microbes, including pathogens that regulate plant community dynamics, decomposers involved in nutrient cycling, and mutualists that enhance plant performance support key ecological functions such as productivity (Van Der Heijden et al., 2008). For example, mycorrhizal fungi benefit plants by increasing nutrient uptake and decreasing abiotic stress and susceptibility to pathogens (Tedersoo et al., 2020). Consequently, advancing scientific research on the synergistic relationship between the soil microbiota and ecological restoration practices is crucial for advancing global restoration (Gnacadja & Vidal, 2023) and is one of the 10 key strategies to meet the targets of the UN Decade on Ecosystem Restoration (Aronson et al., 2020).

One challenge commonly observed in restorations is that certain native plant species often fail to recolonize sites naturally, making them important targets for reintroduction. Here, we refer to these targets as conservative species, which are typically restricted to high‐quality remnant sites and sensitive to human‐mediated degradation (Spyreas, 2019; Swink & Wilhelm, 1994). Although several interacting mechanisms likely drive reintroduction success (De Vitis et al., 2022), soil microbes may be a key factor in this phenomenon. For example, one study found that conservative plant species were more dependent on mycorrhizal fungi and had higher habitat specificity than less conservative species (Bauer et al., 2018). To combat the low reintroduction success rate, inoculating plants with whole soil containing plant mutualists such as mycorrhizal fungi is increasingly applied in restorations to improve plant growth and establishment (Koziol et al., 2022; Middleton & Bever, 2012), yet the effects of soil inoculation on plant establishment vary widely among studies, with positive, neutral, and negative effects reported (Gerrits et al., 2023; Herzberger et al., 2015; Hugron et al., 2020). For example, the microbial composition of soil inoculum, plant community successional stage, and soil P concentrations have all been shown to govern target plant responses to soil inoculation (Neuenkamp et al., 2019). Additionally, studies utilizing soil inoculation for plant reintroductions tend to occur in severely disturbed sites with profoundly altered soil properties (e.g., ex‐arable fields and mine sites), whereas less is known on the role of the soil microbiota in native plant recovery when restoring ecosystems with less disturbed soils.

One factor that may affect the establishment of conservative plant species, which tend to be slower‐growing, longer‐lived, and more dependent on soil symbionts than generalist species, is the timing of reintroduction events (Bauer et al., 2015; Veldman et al., 2015). Abiotic and biotic conditions are predicted to be more favorable for the establishment of conservative species later in restoration (Holl et al., 2022), in part because soil symbionts facilitating plant establishment may only be found in later‐successional sites (Middleton & Bever, 2012). Alternatively, early restorations may lack the establishment barriers potentially encountered later in restoration, such as competition with established vegetation or soil legacies from early‐arriving species (i.e., priority effects, Weidlich et al., 2021), but could be deficient in important microbial mutualists required by rare, conservative plant species.

Additionally, specific restoration practices can influence both soil microbial communities (SMCs) and the establishment of conservative plants in restored sites. For example, the use of fire for habitat management can select against heat‐ or disturbance‐sensitive microbes such as soil‐borne fungi, which are often more sensitive to heat and fire than bacteria (Certini et al., 2021; Hermans et al., 2017; Pressler et al., 2019). Prescribed burns can also affect restored SMCs indirectly through changes in the relative abundance of fire‐sensitive host plants, which may affect the species composition and relative abundance of mutualists (Certini et al., 2021; van Der Heijden et al., 2015). Thus, the timing of reintroducing plants relative to the timing of the onset of prescribed burning could influence the outcomes of SMC assembly and plant reintroduction success.

Oak‐dominated ecosystems that occur across much of the eastern and central United States have undergone dramatic shifts in species composition and structure due to human land‐use activities, invasive species, and alterations in disturbance regimes (Hanberry et al., 2018, 2020). In temperate forests, herbaceous species represent *c*. 80% or more of the plant diversity, including a large proportion of rare species, and contribute significantly to nutrient cycling and overall ecosystem function (Gilliam, 2007). However, fire exclusion has led to encroachment of fire‐intolerant tree species (e.g., *Acer*) that decrease both understory light availability and the abundance and diversity of herbaceous plant species (Nowacki & Abrams, 2008). Restoration of oak ecosystems usually involves prescribed burning or a combination of burning and canopy disturbance to control woody vegetation and encourage passive recovery of a diverse herbaceous flora (Hanberry et al., 2020). Although native plant species richness and abundance often increase after fire or thinning (Lettow et al., 2014; Maginel et al., 2019; Peterson et al., 2007; Vander Yacht et al., 2020), restored oak woodlands often lack conservative species in the understory (Kaul et al., 2023; Reid et al., 2020), leading to questions about whether SMCs can, in part, explain the lack of conservative plant species in restored sites. To date, few studies have explored the relationship between restoration age, the soil microbial communities, and reintroduced plants in woodlands.

In this study, we used an oak woodland restoration chronosequence to study the relationship between restoration age, soil microbiota, and three conservative forb species. Our first objective was to characterize habitat properties and SMCs across the chronosequence. The sites vary in the time since the onset of prescribed burning and thinning, and thus, we expected them to vary in soil chemistry, soil microbial composition, and overall tree density and composition. Our second objective was to test the responses of three conservative forb species to whole‐soil inoculations with soil collected from young, intermediate, or old restored oak woodlands. We expected plant growth to differ in response to inoculation with soil from sites of different restoration ages due to predicted variation in soil microbial and chemical properties. Lastly, we compared soil microbial community composition between the original inocula and the soil after conditioning by each of the focal plant species to determine whether SMCs vary according to plant species, inoculum source, or both. We expected each focal plant species to develop unique core soil microbiota when grown in different inoculum and to exhibit greater growth in inoculum with increased abundance of microbial mutualists and reduced abundance of pathogens.

## 2 METHODS

### 2.1 Study site characterization and soil collection

**FIGURE 1:**
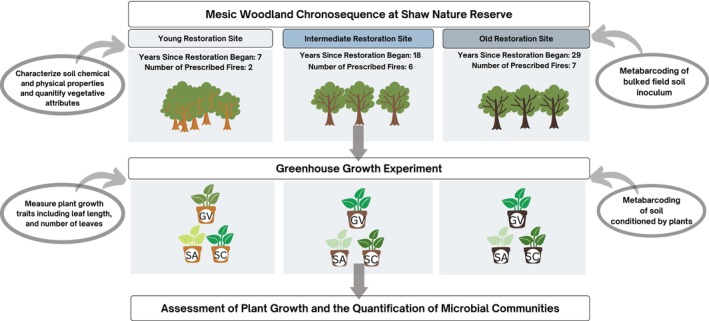
Experimental design. Stages of the experiment that examined the effects of whole‐soil inoculum from an oak woodland restoration chronosequence on plant growth of three native herbaceous species: (GV: *Geum virginianum*, SA: *Solidago arguta*, and SC: *Solidago caesia*).

In September 2019, we established four experimental blocks (20 m^2^) in each of three mesic oak woodland sites at the Missouri Botanical Garden's Shaw Nature Reserve (SNR; 38°28′55" N, 90°49′28″ W), in eastern Missouri, U.S.A. These sites have similar land‐use histories (i.e., tree cutting and grazing, but no known agricultural tilling), soil type (Ultisol silt loam), and span a gradient of time since the onset of restoration to control woody encroachment with prescribed burning and mechanical thinning (Figure 1). The young restoration has been actively restored for 7 years and experienced three prescribed burns, the intermediate restoration has been actively restored for 18 years and experienced six prescribed burns, and the oldest has been actively restored for 29 years and experienced seven prescribed burns. The most recent prescribed burn across all sites occurred at least 1 year before soil sampling. To isolate the effects of restoration age on soils and vegetation while minimizing differences in abiotic and biotic properties caused by topographic variation, all blocks were positioned on N‐NE‐E facing aspects with similar slopes (10%–30%) and distances from slope drainages (5–25 m). These blocks served as the source of soil inoculum for the greenhouse experiment (Section 2.2).

To determine whether restored sites differed in vegetation properties, we quantified the basal area of each tree species in the canopy (DBH ≥ 10 cm) and midstory (height ≥ 1.5 m and DBH < 10) within 0.1 and 0.05‐ha plots, respectively, oriented from the center of each block (four blocks per restoration site). Additionally, in four 1‐m^2^ quadrats in each block (*n* = 16 subplots per restoration site), we recorded percent cover of all herbaceous and woody species (<1.5 m tall) (Full list of vegetative density available in Table S1). To assess soil chemical and physical properties, we randomly collected 10 soil cores (15‐cm depth) in each of the 12 blocks (*n* = 40 soil cores per restoration site) (120 soil cores/block), and quantified 14 chemical and physical soil properties using methods outlined in Albrecht et al. (2022) (also see Table S2).

### 2.2 Greenhouse experimental design

To test the hypothesis that plant performance would differ among the three whole‐soil inoculum treatments, we conducted a controlled greenhouse experiment in April 2019 with three species of perennial woodland herbs: *Geum virginianum* (Rosaceae), *Solidago arguta* (Asteraceae), and *Solidago caesia* (Asteraceae). These species are currently absent in long‐restored oak woodlands at Shaw Nature Reserve and are considered conservative species based on their coefficient of conservatism scores being ≥7 in Missouri (Ladd & Thomas, 2015). The coefficient of conservatism (or C‐scores) is an index ranging from 0 to 10, representing a species' tolerance of environmental degradation, or its fidelity to intact remnant or long‐restored habitats, as determined by local botanical experts (Swink & Wilhelm, 1994). Species with higher C‐scores are less tolerant of degradation and increasingly restricted to high‐quality sites. Our focal species are members of genera known to associate with AMF (Soudzilovskaia et al., 2020).

We obtained seeds of each plant species from wild‐sourced accessions from the Missouri Botanical Garden's seed bank or living collections. We surface‐sterilized seeds with 70% ethanol (45 s) followed by a soak in a 30% sodium hypochlorite solution (120 s) and then rinsed them in distilled water. Seeds were cold stratified on twice‐autoclaved, sterilized sand in Petri dishes at 5°C, with a 12/12 h photoperiod. After stratification, seeds were sown into quart‐sized pots filled to 80% capacity with topsoil sterilized twice by autoclave. We used a custom‐designed peat‐free topsoil mix to mimic the silt loam forest soils at our study site (sand: 30%, silt: 45%, clay: 25%; pH: 5.7).

In June 2019, we collected 1.89 liters of soil (15 cm depth) from four blocks in each of the three restoration sites, and then pooled and homogenized the soil from each of the four blocks within a site (7.56 L of soil per restored site) to create three unique composite bulked field soil samples, which served as our inoculum in the greenhouse experiment (Gundale et al., 2017). We sterilized tools with 70% ethanol before sampling each site. Soil samples were placed on dry ice in the field (<1 day) and then frozen (−20°C) for 7 days until seedling inoculation. A portion of the bulked soil innocula remained frozen for 27 months until DNA extraction and sequencing (see Section 2.3 below).

Each sterile pot was then inoculated with 10 mL of the bulked live field soil (approx. 1% by volume) sourced from one of the three restored sites, a standard approach in plant–soil feedback studies (Pernilla Brinkman et al., 2010). We planted 3–5 stratified seeds directly into the inoculum and then topped off pots with small layer of sterilized soil. Pots were thinned to 1 plant per pot, as needed. In total, we established 270 pots to test for effects of woodland chronosequence inoculum on plant growth (3 soil inoculum treatments × 3 species × 30 replicates). We arranged pots in a completely randomized design in the greenhouse and watered them daily to field capacity. Because these plants were also used in a subsequent experiment to test the effects of soil inoculation on field performance, we could not perform destructive measurements of growth and development. Instead, we measured final leaf length and final number of leaves as a proxy for overall growth and development after 12 weeks, prior to placing plants into the field for a subsequent study. Leaf length has been shown to be correlated with total leaf area and leaf weight (Karimi et al 2009; Neuenkamp et al., 2019), which are often used as proxies for plant growth. Additionally, number of leaves has been used as a reliable proxy for early growth rate in plants (Kaur et al., 2017). The ruler used for measurements was sterilized and the recorder's gloves were changed across treatments.

### 2.3 Soil sample collection, DNA extraction, and sequencing to assess microbial communities

For DNA metabarcoding, we collected approximately 15 mL of soil from seven random pots per treatment at the end of the 12‐week growing period. Samples included field soil‐inoculated pots without plants (3 inocula types × 7 replicates, *n* = 21) as well as pots from each plant in each inocula type (3 plant species × 3 inocula types × 7 replicates, *n* = 63), for a total of *n* = 84 samples. In pots with plants, our samples included soil at the soil‐root interface (rhizosphere) and fine root fragments (hereafter called “plant‐conditioned soil”). Soil samples were frozen for 24 months at −20°C until DNA extraction.

For DNA extraction, library preparation, and DNA sequencing, we followed protocols previously described in detail in Swift et al. (2018), with some modifications. Briefly, DNA was extracted from all samples (bulked field soil inocula and plant‐conditioned soil) using a CTAB protocol (cetrimonium bromide; Doyle & Doyle, 1987), which was modified with smaller lysis and wash volumes and an added 95% ethanol wash. For each batch of DNA extractions, we included two no‐template controls (NTCs) to measure potential contamination introduced during laboratory processing. Each sample (and NTC) next underwent separate PCR amplifications for three target loci: bacterial 16S, fungal ITS, and fungal 5.8 s, the latter of which is specifically designed to increase the coverage of arbuscular mycorrhizal fungi (AMF), which does not amplify well using standard fungal ITS primers (See Table S3 for a list of PCR primers). Each set of PCR primers included a F or R Illumina adaptor sequence added to the 3′ end for use in a second indexing PCR (see below). The amplicons for the three target loci for each sample were quantified using a QUBIT Fluorometer and then pooled in equimolar ratios. This pooled PCR product for each sample was used as template for a second PCR to add sample‐specific indexes using the NEXTERA XT index kit. The indexed PCR products for each sample and NTC were quantified and pooled in equimolar ratios. Finally, the pooled sets of 90 sample libraries, including seven replicates for each inoculum and each soil–plant combination (84), four extraction NTCs, and two PCR NTCs all were sequenced on an Illumina® MiSeq using the MiSeq Reagent Kit v3 (600‐cycle; Illumina, Inc.) at the Duke Center for Genomic and Computational Biology.

### 2.4 Processing of microbial DNA sequences

We conducted all sequence processing in QIIME2 (v.2‐2022.2). We first used Cutadapt 2020.2.0 to trim Illumina primers, indices, and retained primers from sequences. Additionally, because our sequences were pooled per sample, we used cutadapt to split sequences by primer, creating two separate feature artifacts for sequences containing 16S and ITS/AMF fungal reads (Martin, 2011). For each of the two aforementioned datasets, we filtered and trimmed reads to a uniform length using the divisive amplicon denoising algorithm (DADA; v.1.10.1; Callahan et al., 2016) using default parameters and processed sequences into amplicon sequence variants (ASVs). Next, we assigned taxonomy to each ASV for both datasets using the QIIME feature classifier function. 16S sequences were taxonomically assigned using a trained SILVA classifier, which targets Bacteria and Archaea (Pruesse et al., 2007). Fungal sequences were taxonomically assigned using the UNITE classifier, which broadly targets Fungi (Nilsson et al., 2019). We identified 13,987 bacterial 16S ASVs and 7522 fungal ASVs. The average number of reads per sample across all primers was 58,004.

### 2.5 Statistical analyses

#### 2.5.1 Soil, vegetation, and microbial communities across the restoration chronosequence

All statistical analyses were performed in R v.4.2.2. To determine how total tree species composition (combined canopy and midstory) and herbaceous species composition differed across restoration sites, we first conducted a permutational multivariate analysis of variance (PERMANOVA) with Bray–Curtis dissimilarity with 999 permutations using the adonis2 function in the package “vegan” (Oksanen, 2022), as well as a dispersion test using the function permdisp in the package vegan. We visualized data via ordination by non‐metric multidimensional scaling (NMDS), reducing the data to two dimensions. If PERMANOVA results revealed significant differences between restoration sites, we then utilized the function “envfit” in the package BiodiversityR (Kindt & Kindt, 2019) to determine which species contributed to differences among sites.

To determine if soil chemical properties varied across the chronosequence, we conducted a principal component analysis (PCA). We first performed a correlation test using the R package “corrr” to remove variables with high collinearity. After removing three highly correlated variables (Sand %, Clay%, and Na concentration *r* > .8), we performed a PCA using the function *prcomp* with 11 soil variables collected from the 12 blocks, which were standardized for analysis (Table S2; Figure 1). Additionally, we conducted separate one‐way analysis of variance (ANOVA) models for each of the 11 soil variables (Table S2). Variables were log‐transformed as needed to meet the assumptions of ANOVA. When main effects were significant (*p* < .05), we conducted Tukey HSD post‐hoc comparisons to test for differences among restoration sites.

To determine whether Shannon diversity of SMCs differed with restoration age, we first calculated Shannon index using the “estimate_richness” function in the package phyloseq (McMurdie & Holmes, 2015). Once richness was calculated for each sample, we performed separate one‐way ANOVA tests for fungal and bacterial ASVs. Additionally, we calculated Bray Curtis dissimilarity using the function “braydist” and “metaMDS” from the package *vegan*. To assess whether the composition of fungal and bacterial communities differed with restoration age, we applied a PERMANOVA. Compositional variation in microbial communities was visualized with NMDS based on Bray–Curtis dissimilarity. Lastly, we calculated similarity percentages by utilizing the SIMPER function in the “vegan” package to assess the percent contribution of each bacterial or fungal ASV to Bray–Curtis dissimilarity. For fungal taxa with a SIMPER contribution over 1%, we assigned functionality using FUNGuild (Nguyen et al., 2016) and then recalculated the total percentage contribution by functional group.

#### 2.5.2 Plant growth in response to soil inoculation treatment

To determine whether the final growth of the three plant species varied in response to soil inoculum treatment, we used a linear model to assess the effect of soil inoculum on final leaf length at week 12 for each of the three focal plant species separately. We then computed least squares means using the function *lsmeans* (Lenth, 2016), and tested for pairwise differences in leaf length among soil treatments using Tukey adjusted *p*‐values. We compared number of leaves at week 12 by using a generalized linear model with binomial distribution with number of leaves as a function of soil type for each species separately.

#### 2.5.3 Changes in soil microbial communities after soil conditioning by forb species

First, we calculated Shannon diversity using the same methods previously described from the package phyloseq. We then used linear models to test how soil microbial alpha diversity differed within and among plant species in response to soil inoculation treatments. Separate models for bacterial and fungal taxa were constructed where Shannon diversity of the given taxa was included as a response variable, and soil inoculum, plant species, and their interaction were included as fixed effects. Additionally, we calculated Bray–Curtis dissimilarity using the function “metaMDS” from the package vegan. Next, we used PERMANOVA (999 permutations) to assess the effects of soil inoculum, plant species, and their interaction on bacterial and fungal Bray–Curtis dissimilarity (*p* < .05). Additionally, we performed an indicator species analysis (Dufrêne & Legendre, 1997) to compare the principal microbial taxa associated with plants grown in the three inoculated soils, and a differential abundance analysis using phyloseq_to_deseq2 function in the package “phyloseq” to reveal significant differential abundance of microbial families as a function of soil inocula and plant species (McMurdie & Holmes, 2015). We performed two pairwise comparisons for bacterial taxa and fungal taxa to assess SMC of young restored sites compared to older sites: (1) plants grown in inocula from the young restored site compared to plants grown in inocula from the old restored woodland, and (2) plants grown in inocula from the young restored woodland compared to plants grown in inocula from the intermediate restored woodland. Though three pairwise comparisons were possible, we focused on young vs intermediate and young versus old due in large part to our findings that plants grown in young soil outperformed those in both intermediate and old soil. We corrected for multiple tests with a Bonferroni correction (*p* = .025). We also visualized abundance with differential abundance heatmaps and relative abundance plots for each plant‐inocula combination.

To determine how key fungal guilds implicated in plant performance shifted when comparing bulked field soil inocula and after plants conditioned the soil, we retrieved a subset of fungal taxa that were taxonomically annotated to Family (taxonomic level 5). This subset of data represented 608 ASVs out of 7034. We then performed SIMPER analysis on this subset of taxa across restoration ages for both the bulked field soil inocula and plant‐conditioned soil from the greenhouse experiment to examine changes in the relative abundance of microbial taxa. Next, we assigned functional guild to taxa significantly contributing (>1%) to Bray–Curtis dissimilarity using probable FUNGUILD assignments (Nguyen et al., 2016) and clustered contributions by assigned guild.

## 3 RESULTS

### 3.1 Soil and vegetation properties and microbial communities across the restoration chronosequence

**FIGURE 2:**
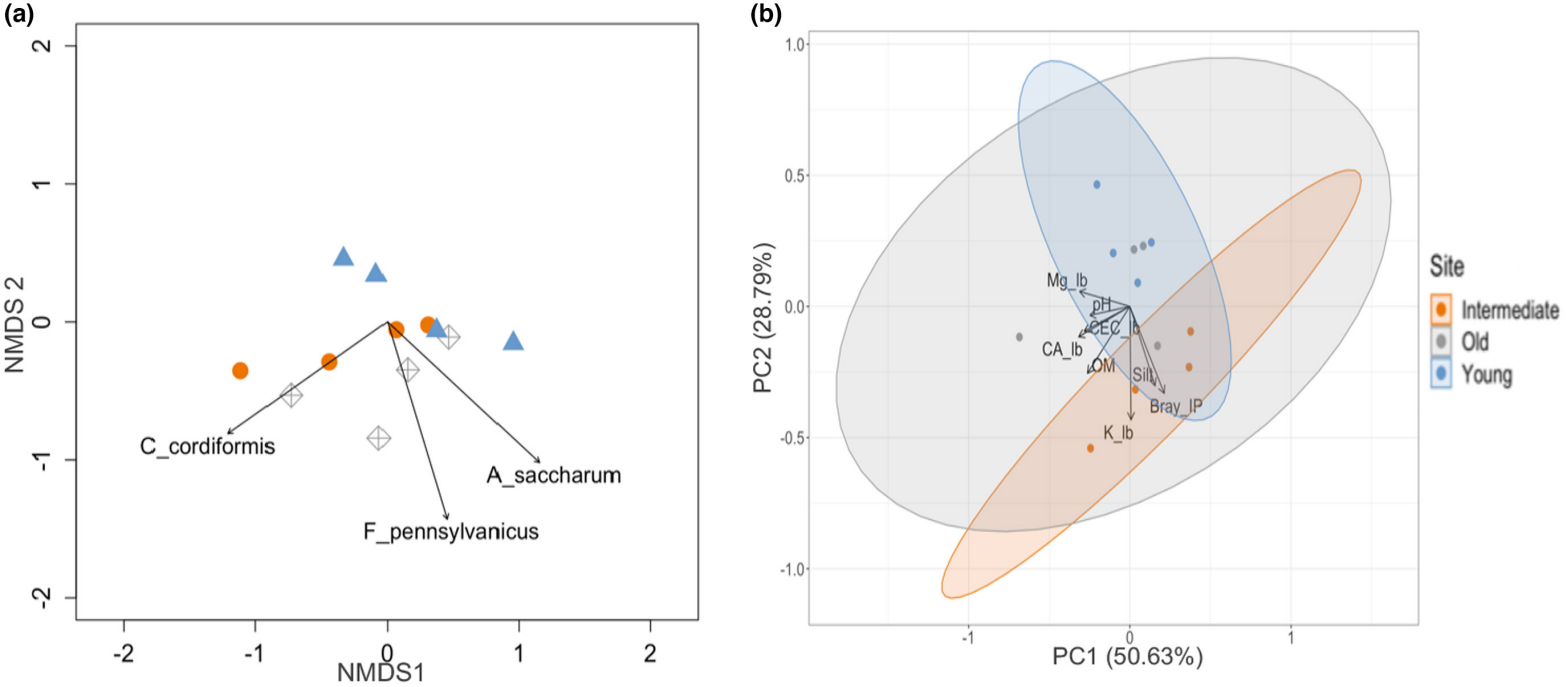
Ordination plots characterizing tree composition and soil properties of restoration chronosequence. (a) NMDS of tree species composition data standardized by species maximum. Tree species with significant differences in abundance (*p* < .05) are included as ordination axes. (b) Principal component analysis assessing soil physical and chemical properties in each restoration site in the chronosequence. PC1 and PC2 account for 79.42% of the total variance observed. Points are color‐coded by unit, Young = Blue, Intermediate = Orange, and Old = Gray.

When assessing vegetative and soil properties, we find some differences between the restoration sites. Tree species composition varied between plots across the restoration chronosequence (PERMANOVA, *F* = 3.901, *p* = .001; permdisp dispersion test, all dispersion values *p* > .05). The post hoc envfit test revealed that the abundance of three tree species contributed significantly to differences among sites: *Acer saccharum* (*p* = .035) and *Fraxinus pensylvanicus* (*p* = .045) in young and *Carya coordiformis* (*p* = .051) in the old restoration site (Figure 1). Herbaceous species composition did not differ across restoration sites (*F* = 2.189, *p* = .299). Soil phosphorus (Bray‐IP) availability and potassium (K/Kg/Ha) concentrations varied significantly among restored sites (*F*_3,11_ = 9.649, *p* = .006; *F*_3,11_ = 19.83, *p* < .001 respectively). Soils in intermediate and older restored woodlands contained greater concentrations of P and K than the young restored woodland (Figure 2; Table S2). However, other soil chemical properties, including pH and organic matter, did not differ across the chronosequence (Table S2).

**FIGURE 3:**
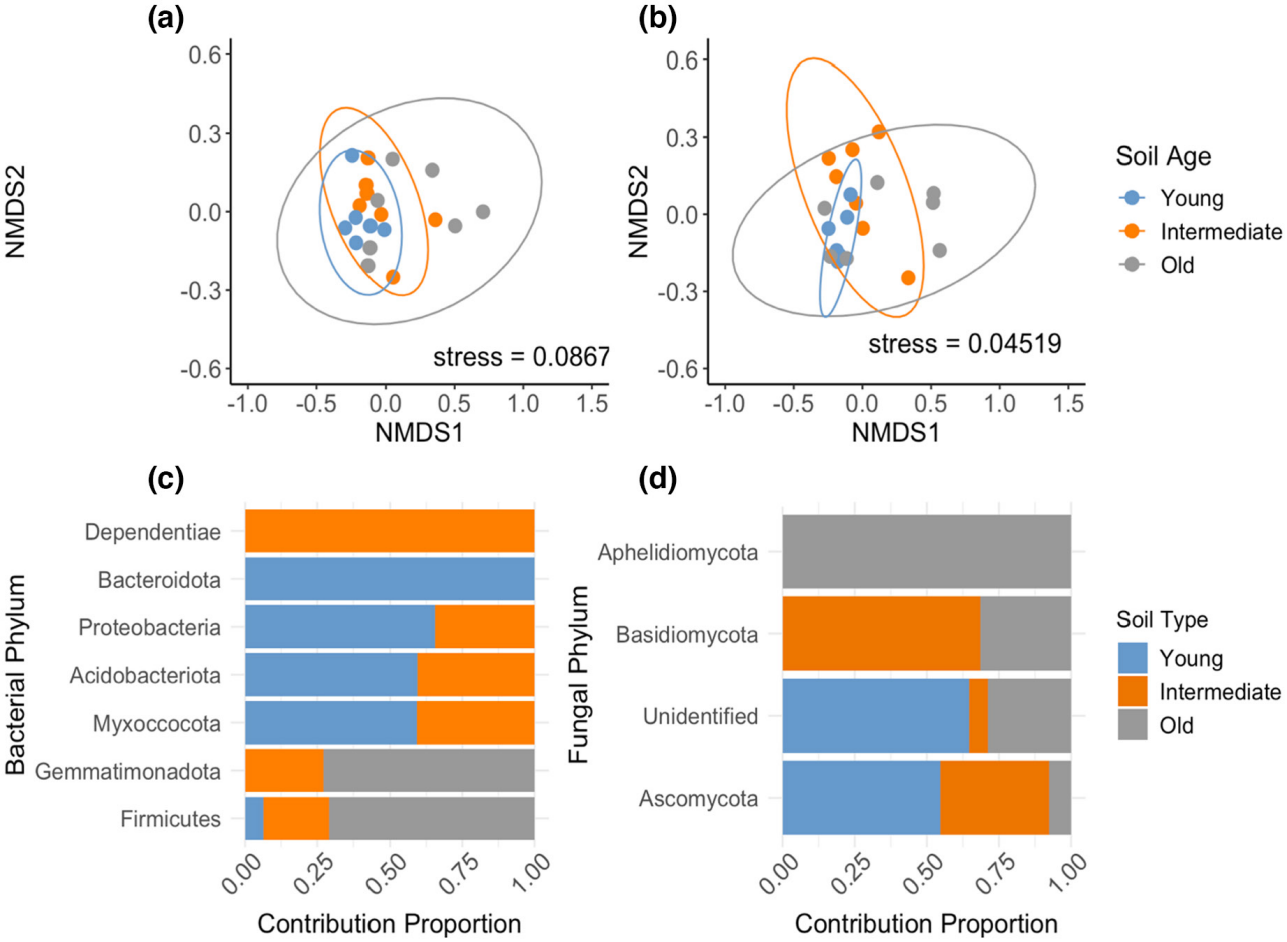
Microbial community composition in soils from bulked field soil inocula collected in young, intermediate, and old restored oak woodlands. Non‐metric multidimensional scaling (NMDS) of (a) bacterial and (b) fungal community composition, and main contributors (>1%) to Bray–Curtis dissimilarity based on similarity percentages (SIMPER) of (c) bacterial and (d) fungal phyla. The bars represent the proportion of contributions by phyla from each soil inoculum. Each point represents a replicate of each inoculum with 95% confidence ellipses around the centroid.

In the bulked field inocula, neither fungal nor bacterial Shannon diversity differed with restoration age (*p* = .051; *p* = .348, respectively). In contrast, bacterial (*p* = .002) and fungal (*p* = .032) microbial community composition significantly differed with restoration age (PERMANOVA, Figure 3). According to the similarity percentage procedure (SIMPER) analysis, Firmicutes (Figure 3c) and fungal Ascomycota (Figure 3d) had the highest percentages of differentiation across the chronosequence. Other bacterial phyla with important contributions to Bray–Curtis dissimilarity (phyla contributing >1%) across restoration sites included: Gemmatimonadota, Acidobacteria, Myxoccocota, and Proteobacteria, with the proportion of contribution by these taxa varying among soil ages (Figure 3c). Other fungal phyla with important contributions (>1%) included: Basidiomycota, Aphelidiomycota, and unidentified fungal phyla, with the relative contribution varying across the chronosequence (Figure 3d).

### 3.2 Plant growth response to soil inoculum treatments

**FIGURE 4:**
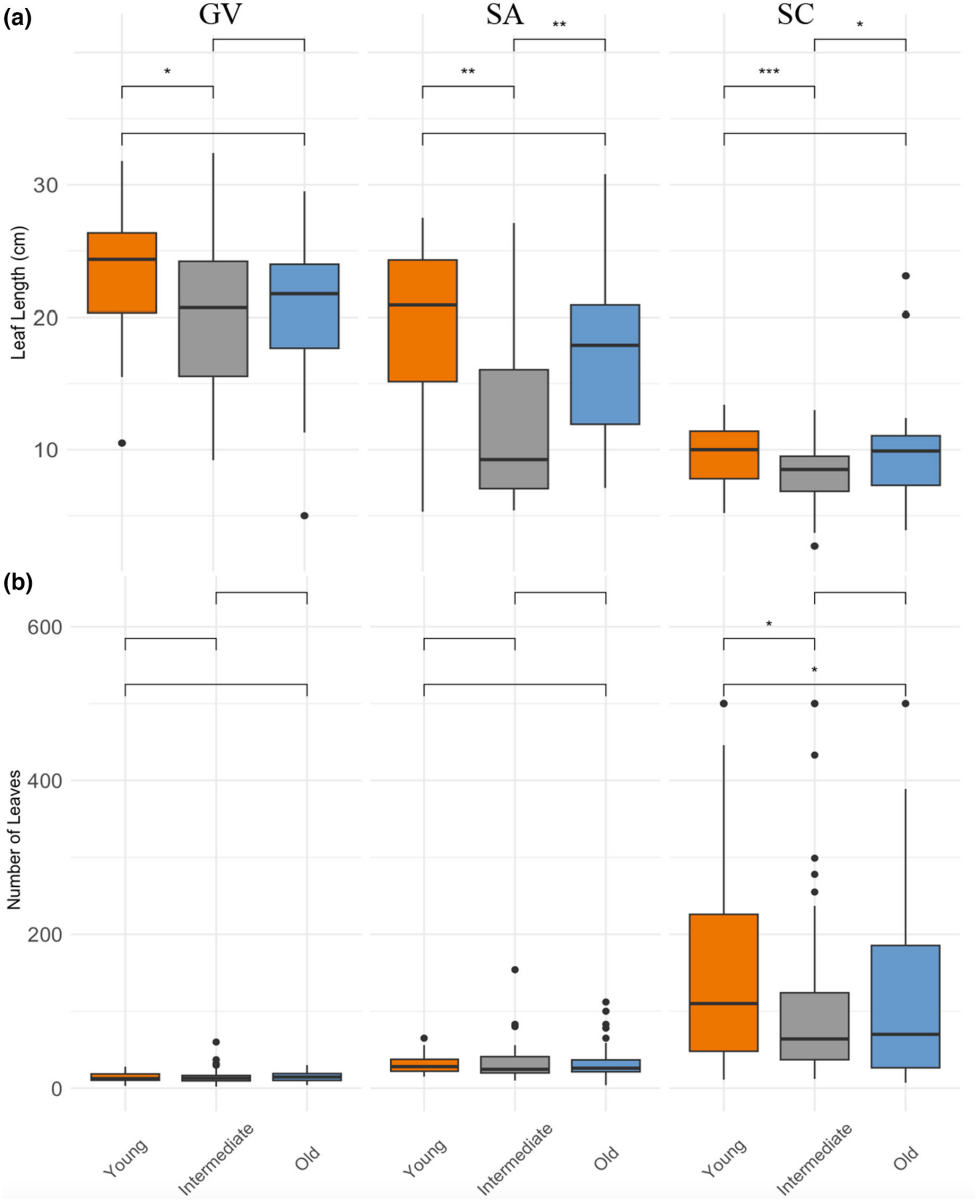
Differences in (a) Leaf length and (b) Number of leaves after 12 weeks of growth for three focal species (GV: *G. virginianum*, SA: *S. arguta*, SC: *S. caesia*) grown in soil inoculated from young, intermediate, and old woodland restoration sites at Shaw Nature Reserve.

After 12 weeks, the effect of inoculation on leaf length and number of leaves varied significantly between soils for each of the three herbaceous species (Figure 4). Each plant species developed significantly longer leaves when grown in soil inoculum from the younger restoration site compared to the intermediate restoration site. Additionally, *S. arguta* and *S. caesia* grew longer leaves in soil inoculated from the intermediate site compared to old (*p* < .001 and *p* < .001, respectively). In *G. virginianum*, plants tended (*p* = .067) to produce longer leaves when grown in inoculum from young relative to the old restoration site (Figure 4). When considering number of leaves, only *S. caesia* grew significantly more leaves when grown in different bulked field soil inocula (*p* = .029; Figure 4).

### 3.3 Changes in soil microbial communities after soil conditioning by forb species

After conditioning by plants, we find that bacterial and fungal Shannon diversity did not significantly differ across soil treatments (*p* > .05 for each contrast). Additionally, community composition in conditioned soils differed significantly across plant species and soil inoculum treatments, but the interaction was not significant for bacteria (PERMANOVA; plant species: *p* = .001, *F* = 2.348, soil age: *p* = .024, *F* = 1.363, plant × soil *p* = .088, F = 1.134) or fungi (plant species: *p* = .042, soil age *p* = .010, plant × soil: *p* = .86; Figure S1). However, indicator species analyses revealed unique soil microbiota across plant species and soil inoculum treatments (Table S4). When comparing the number of unique ASVs associated with each conditioning treatment (plant species × soil inoculum), we found that plant species associate with unique taxa when grown in the same soil inoculum treatment, and conspecifics cultivate unique core taxa when grown in soils with inoculum from different restoration ages. This further suggests that plants altered initially identical soils to create distinct SMC.

**FIGURE 5:**
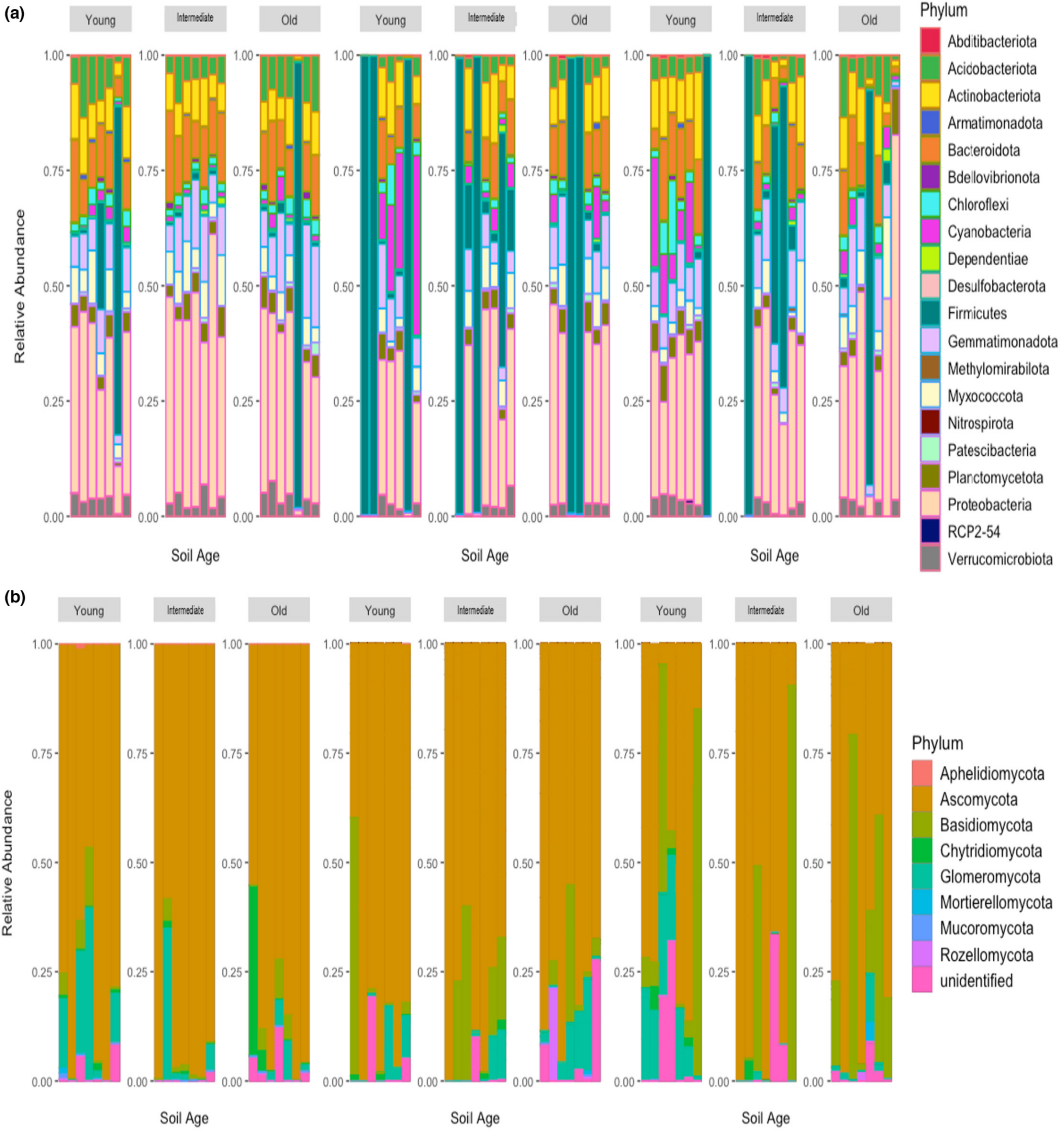
Relative abundance plots of top 20 (a) bacterial and (b) all fungal taxa across soils conditioned by three focal plant species (GV, SA, SC) sorted by phyla.

After conditioning by plants, we also observed unique patterns in SMCs corresponding to the restoration age of the inoculum. After conditioning, microbial communities in the younger restored soils had greater abundance of plant growth‐promoting microbiota including Glomeromycota and Cyanobacteria, and lower abundance of Ascomycota, Basidiomycota, and Firmicutes relative to those from intermediate and older restored soils (Table 1; Figure 5; Figure S2). Similarly, plants inoculated with soil from the intermediate restoration showed SMCs with greater ASV abundance of Ascomycota and Basidiomycota and lower abundance of unknown fungi, Chytridiomycota, Firmicutes, Cyanobacteria, and Proteobacteria than those inoculated with old conditioned soils (Table 1; Figure 5; Table S5; Figure S2).

**TABLE 1:** Similarity percentage analysis (SIMPER) of microbial phyla from conditioned soil across all plant species.

Young vs. Old soil				
Domain	Phylum	Total (%)	Young (%)	Old (%)
Fungi	Unknown	24.9	7.90	17.00
Fungi	Ascomycota	20.3	8.10	12.20
Fungi	Glomeromycota	2.7	2.70	0.00
Fungi	Basidiomycota	2.4	0.00	2.40
Fungi	Chytridiomycota	1.1	0.00	1.10
Bacteria	Firmicutes	13.8	4.88	8.97
Bacteria	Cyanobacteria	2.4	2.35	0.00
Bacteria	Myxococcota	1.0	0.00	1.03

Young vs. Intermediate soil				
Domain	Phylum	Total (%)	Young (%)	Intermediate (%)
Fungi	Ascomycota	28.4	5.4	23.00
Fungi	Unknown	18.8	15.50	3.30
Fungi	Glomeromycota	2.4	2.40	0.00
Fungi	Basidiomycota	2.3	0.00	2.30
Bacteria	Firmicutes	15.7	4.38	11.27
Bacteria	Cyanobacteria	2.6	2.63	0.00
Bacteria	Myxococcota	1.1	1.05	0.00

Intermediate vs. Old soil				
Domain	Phylum	Total (%)	Intermediate (%)	Old (%)
Fungi	Ascomycota	30.3	21.90	8.40
Fungi	Unknown	17.0	3.00	14.00
Fungi	Basidiomycota	4.7	2.40	2.30
Fungi	Chytridiomycota	1.0	0.00	1.00
Bacteria	Firmicutes	13.2	5.70	7.50
Bacteria	Proteobacteria	1.2	0.00	1.22
Bacteria	Myxococcota	1.2	0.00	1.20

*Note*: SIMPER analysis calculates the percentage that particular taxa contribute to Bray–Curtis dissimilarity, revealing the drivers of Beta diversity.

Some microbial taxa exhibited consistent patterns across all plant species within an inoculation treatment, whereas others showed plant species‐specific patterns of microbial abundance. For example, Burkholderiaceae, a bacterial family in the phylum Proteobacteria, was significantly more abundant in soils after all focal plant species were grown in inoculum from the young restoration site compared to inoculum from the old restoration site (Figure 5). In contrast, Polyangiaceae, a family in the phylum Myxococcota, was significantly more abundant when *S. caesia*, but not the other focal plant species, was grown in inoculum from the young restoration relative to the other inoculum treatments (Figure 5). Similarly, Glomeraceae, which includes AMF, was marginally more abundant after *S. caesia* was grown in young inoculum, whereas Ascobolaceae was significantly abundant only when *G. virginianum* was grown in old soil inoculum.

**FIGURE 6:**
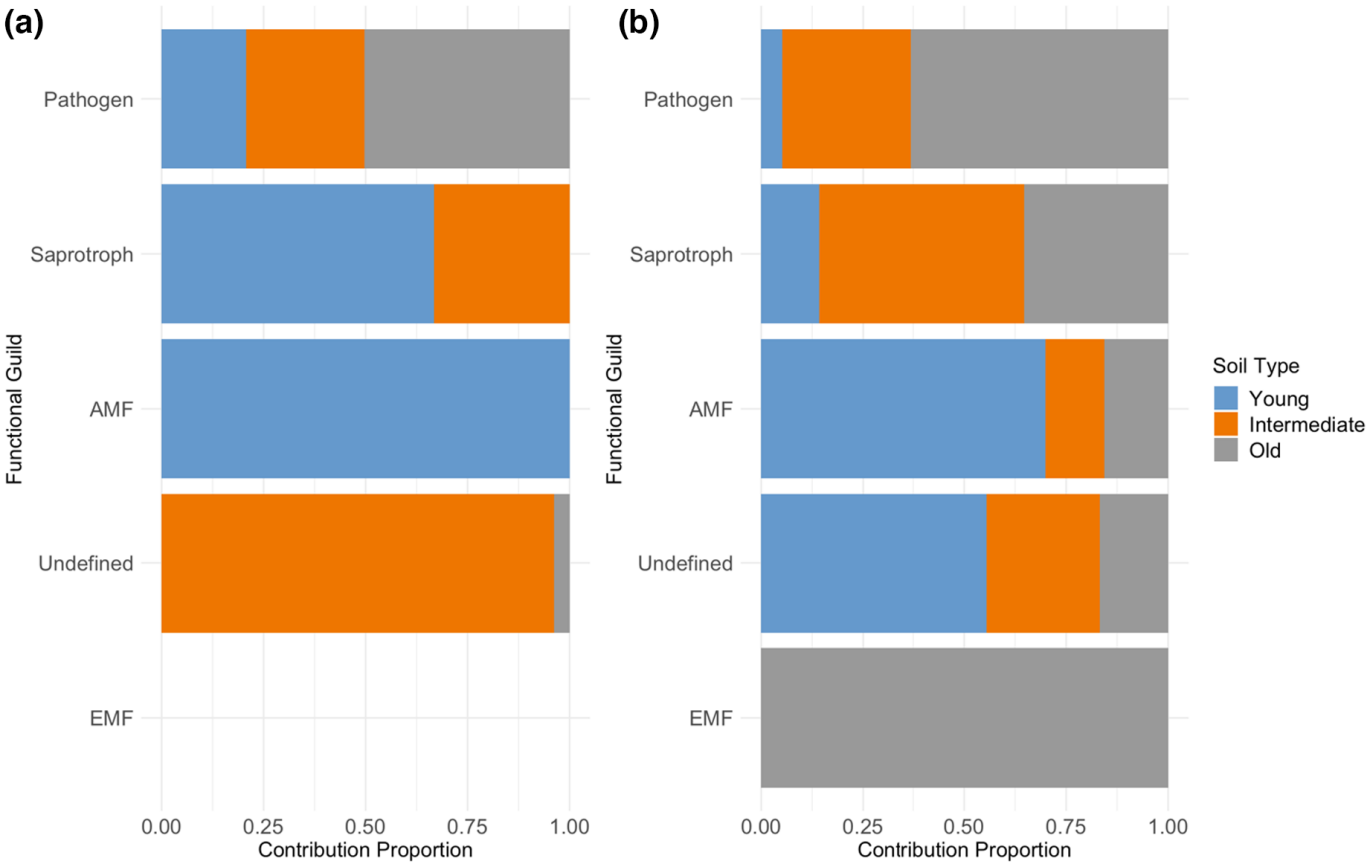
Results of the SIMPER analysis of a subset of functionally annotated fungi from (a) initial soil inoculum and (b) post‐growth conditioned soil. Bars represent the sum of the contribution by guild in (a) all inocula and (b) across all plant species grown in each inoculum.

Lastly, the relative proportions of fungal guilds diverged after plants conditioned soils from each restoration site (Figure 6). For example, AMF exhibited the greatest relative abundance after the focal plant species were grown in inoculum from the young restoration, whereas pathogens and ectomycorrhizal fungi (EMF) exhibited greater relative abundances when the old restoration served as the inoculum source (Figure 6).

## 4 DISCUSSION

Our study examined changes in soil microbial communities (SMCs) across a restoration chronosequence and tested whether whole‐soil inoculum from sites with variable restoration ages affected seedling performance of three native forbs. The composition of SMCs varied across the chronosequence, with compositional shifts consistent with the expected responses of soil microbiota to repeated burning. Seedling performance of herbaceous species varied in response to whole‐soil inoculum treatments, with enhanced growth in soil inoculum from the youngest restored woodland, which contained a greater abundance of AMF and a lower abundance of pathogens. These results suggest that conservative forb species in oak woodland are sensitive to variation in SMCs and that soils in early stages of oak woodland restoration are likely more suitable for the establishment of difficult‐to‐reintroduce conservative species.

We hypothesized that increased prescribed burning would alter above‐ground vegetation structure and soil microbial diversity and composition across a chronosequence of restored oak woodlands. Surprisingly, we observed few discernible differences in both vegetative and soil abiotic properties among the restored woodland sites. As expected, tree species composition varied across restored sites, primarily driven by an increased relative abundance of fire‐sensitive and arbuscular mycorrhizal‐associating tree species in the young restoration site. However, we found little detectable difference in the abundance of mycorrhizal‐fungi (Glomeromycota) across bulked field soil inocula, suggesting that shifts in tree species composition across the restoration gradient may have only a minor influence on bulk soils. Recent studies found that AMF abundance is greatest in rhizosphere soil compared to bulked soils (Wattenburger et al., 2020), indicating the localized impact of plant‐mycorrhizal interactions is strongest within a certain radius of their root. Furthermore, we found plant community composition in the herb layer differed little across the chronosequence. Because our study focuses on mesic oak woodlands in sheltered topographic positions with naturally low light, herbaceous vegetation may be less responsive to repeated burning and thus may impose little influence on site‐wide SMC. This aligns with a recent study in deciduous forests that demonstrated a large increase in herbaceous‐layer diversity resulted in only slight increases in the diversity and biomass of bacterial and fungal taxa while microbial composition remained relatively unchanged (Stefanowicz et al., 2022).

Soil phosphorus (P) and potassium (K) concentrations increased with restoration age, peaking in the intermediate and old restored sites. This is consistent with findings from prior studies that showed post‐fire soil nutrient content, particularly K and P, to be significantly higher post‐burning (Butler et al., 2018; Lekberg et al., 2021) Similarly, oak forests subjected to an increased number of prescribed burns exhibited elevated levels of various soil nutrients, including Na, Ca, K, and P (Scharenbroch et al., 2012). In older restored soils, there is an established link between high P levels and an increase in pathogenic fungi at the expense of symbiotic taxa (Lekberg et al., 2021; Neuenkamp et al., 2019). This complex interplay between soil chemistry and microbial interactions is consistent with the Habitat Hypothesis (Horn et al., 2017; Zobel & Öpik, 2014), which posits that changes in abiotic conditions significantly influence plant‐fungi relationships.

Restoration and management activities, such as haying, thinning, and fire, significantly modify both aboveground and belowground community composition (e.g., Mittal et al., 2019; Ondik et al., 2022). For example, previous studies found that fire selected against disturbance‐sensitive microbial taxa (Certini et al., 2021), depleted plant growth‐promoting bacteria (PGPB) in pine forests (Mittal et al., 2019), and increased the abundance of Firmicutes bacteria, which outcompeted other bacterial taxa, in severely burned regions (Lucas‐Borja et al., 2019). In the present study, soils from the young restored woodland had a significantly greater abundance of Proteobacteria, a phylum containing PGPB (Cox et al., 2022; Pérez‐Valera et al., 2019), whereas soil from the oldest restored woodland showed significantly greater abundance of Firmicutes and Ascomycota. Previous research found that fungal taxonomic composition in recently burned, late‐successional habitats also shifted toward pyrophilic taxa in the Ascomycota and fast‐growing Basidiomycota (Bonanomi et al., 2022). Surprisingly, even though we found greater AM‐associating tree species in the young restored woodland, AMF fungi did not differ in abundance across the chronosequence in bulked field soils, suggesting that AMF may respond neutrally or slowly to low‐intensity, periodic prescribed burns (Beals et al., 2022; Revillini et al., 2022).

The results of our greenhouse experiment suggest that differences in SMC influence plant growth. All three focal plant species developed larger leaves, and one species produced a greater number of leaves, when grown in soil inoculum from the young restored woodland. After plants conditioned soils, we found significantly greater abundance of AMF in the young soil inocula suggesting that plants were more readily colonized by AMF when inoculated with soil from the young restoration. Though we did not detect differences in the abundance of AMF across bulked soil inocula treatments, it is possible that AMF‐associating tree species in the young restoration harbored unique AMF taxa that resulted in increased plant growth and AMF colonization for plants conditioned with young soil inoculum. However, the low resolution of fungal taxa in our metabarcoding data prohibited the taxonomic depth needed to address this hypothesis. Another possibility for the increased plant growth in the young restoration inoculum could be a greater abundance of microbes inhibiting growth in the intermediate and older restorations. For example, old conditioned soil showed an increased abundance of pathogenic fungi (e.g., *Fusarium*), which can possibly inhibit the establishment of AMF and affect plant growth (Ballhorn et al., 2014). Fungal pathogens can increase in abundance after fire, becoming the dominant soil microbial functional group (Pérez‐Valera et al., 2019).

After plants conditioned bulked field‐soil inocula, bacterial and fungal alpha diversity did not vary significantly across restoration treatments. However, we found differences in the functional diversity and composition of SMCs consistent with results from other SMC chronosequence studies (Gellie et al., 2017; Yan et al., 2018), including mine site restorations (Ezeokoli et al., 2020; Van Der Heyde et al., 2020). Additionally, we found shifts in SMC between bulked field soil inocula and plant‐conditioned soils. In the bulked field inocula, mycorrhizal fungal groups containing mutualists, such as AMF, did not significantly contribute to differences in composition or differential abundance. However, after plants conditioned soils, microbial phyla that are known to form mutualistic or pathogenic relationships with plants, including Cyanobacteria, Glomeromycota, and Ascomycota, increased in abundance and shifted in composition.

Consistent with our predictions, each plant species cultivated unique soil microbiota. The microbial communities that developed 12 weeks after inoculation diverged considerably in composition from the bulked field soil inocula and varied among plant species. For example, members of the same genus, which are sometimes used interchangeably in restoration due to their assumed functional equivalence, formed strongly divergent soil microbiota when grown in the same soil type. In young soil inoculum, *S. caesia* cultivated greater abundance of Glomeromycota and Basidiomycota fungi, as well as *Fusarium*, a potentially pathogenic fungal genus in the phylum Ascomycota. In contrast, *S. arguta* produced significantly higher abundance of Proteobacteria in young soil, an important group for soil nitrogen cycling (Ren et al., 2018), and greater abundance of Firmicutes, which may be important for restoration success due to their ability to improve iron reduction (Gupta et al., 2018). Overall, our findings align with previous studies that found plant species identity may be a primary driver of bacterial and fungal community composition (e.g., Brundrett, 2002; Burns et al., 2015). The development of unique SMCs by different plant species means that whole‐soil inoculations could affect important factors such as plant community assembly and species persistence when restoring oak woodlands (Bever et al., 2010).

The differences in the microbial communities and their effects on plant growth could have important implications for restoration practice. Plants grew better when inoculated with soil from young at the restoration site, which suggests that conservative forb species in oak woodland are sensitive to variation in SMCs and that soils in early stages of oak woodland restoration may promote the establishment of difficult‐to‐reintroduce species. These results are counter to the long‐standing hypothesis that “older is better” for reintroduction, where a more well‐established restoration site with an associated late‐successional microbial community is assumed to improve plant growth (Hugron et al., 2020). In contrast, we found that plants are more likely to cultivate greater abundances of soil mutualists and lower abundances of pathogens in younger restored soils, and this may depend on variation in tree species composition across sites. However, further analysis is needed to understand whether our results extend to sites beyond the oak woodland chronosequence studied here. Another relevant question is whether differences in plant growth and microbial communities observed in the greenhouse persist after the focal plant species are transplanted into the field.

Soil microbiota differed in response to each unique combination of plant species and inoculum investigated in the experiment. These results suggest that careful consideration is needed when selecting plant species and inoculum source when planning restorations because they could affect the performance of reintroduced plants and shift soil microbial function. However, additional research is needed to understand the complex relationship between restoration age, plant species performance, and microbiota in restored oak woodlands. Clearly, successful restoration will require a holistic approach (Moreno‐Mateos et al., 2020), pairing knowledge of plant life history characteristics with analyses of the soil microbial community structure, acknowledging that both conservation management practices and soil microbiota synergistically influence restoration outcomes.

## AUTHOR CONTRIBUTIONS

**Rachel A. Brant:** Formal analysis (lead); investigation (equal); visualization (lead); writing – original draft (lead); writing – review and editing (lead). **Christine E. Edwards:** Formal analysis (equal); investigation (supporting); methodology (supporting); writing – review and editing (supporting). **John Leighton Reid:** Conceptualization (equal); data curation (equal); funding acquisition (equal); methodology (equal); writing – review and editing (supporting). **Burgund Bassüner:** Formal analysis (equal); investigation (equal); validation (equal). **Noah Dell:** Data curation (equal); formal analysis (supporting); writing – review and editing (supporting). **Brad Delfeld:** Data curation (equal); writing – review and editing (supporting). **Scott A. Mangan:** Conceptualization (equal); data curation (equal); funding acquisition (equal); investigation (equal); writing – review and editing (supporting). **Victoria de la Paz Bernasconi Torres:** Conceptualization (equal); funding acquisition (equal); investigation (supporting); methodology (supporting); writing – review and editing (supporting). **Matthew A. Albrecht:** Conceptualization (equal); data curation (equal); funding acquisition (equal); methodology (equal); supervision (equal); writing – review and editing (supporting).

## CONFLICT OF INTEREST STATEMENT

Authors declare no conflict of interest.

## Supporting information


Data S1.

## Data Availability

Data are available at https://github.com/rachelbrant/LECdata/.
