# Supplementary material for: Restoration age affects microbial‐herbaceous plant interactions in an oak woodland
Source: Ecol Evol. 2024 May 2;14(5):e11360. doi: 10.1002/ece3.11360 (PMC11066493; doi:10.1002/ece3.11360)
Supplement: Supplementary file 1 — Data S1. [file ECE3-14-e11360-s001.docx]

Supplementary Material: Restoration age alters soil microbial communities and shapes interactions between microbiota and plant growth in an oak woodland. Brant et al 2023

Table S1. Table of ANOVA tests comparing tree density (by genera)

| **Type** | **Genus** | **Df** | **SumSq** | **MeanSq** | **F** | **P** |
| --- | --- | --- | --- | --- | --- | --- |
| Canopy | Acer | 2 | 4872 | 2436 | 30.99 | **5.18E-05** |
| Canopy | Carya | 2 | 190.3 | 95.15 | 0.7011 | 0.5090 |
| Canopy | Celtis | 2 | 14.79 | 7.395 | 9.022 | 0.05390 |
| Canopy | Cornus | - | - | - | - | - |
| Canopy | Diospyros | - | - |  | - | - |
| Canopy | Fraxinus | 2 | 377.0 | 188.5 | 0.951 | 0.4780 |
| Canopy | Juniperus | 2 | 79.32 | 39.66 | 1.91 | 0.2870 |
| Canopy | Platanus | 2 | 4422 | 2211 | 12 | 0.2000 |
| Canopy | Quercus | 2 | 897.0 | 448.4 | 0.35 | 0.7050 |
| Canopy | Ulmus | 2 | 2.090 | 1.046 | 0.0970 | 0.9100 |
| Canopy | Juglans | - | - | - | - | - |
| Canopy | Sassafras | - | - | - | - | - |
| Midstory | Acer | 2 | 207.3 | 103.6 | 30.60 | **5.30E-12** |
| Midstory | Asimina | 1 | 0.6600 | 0.6590 | 0.0586 | 0.8160 |
| Midstory | Carya | 1 | 3.960 | 3.960 | 0.8410 | 0.4110 |
| Midstory | Celtis | 1 | 16.44 | 16.44 | 13.70 | 0.003030 |
| Midstory | Cornus | 2 | 38.93 | 19.46 | 9.270 | 0.0007300 |
| Midstory | Fraxinus | 2 | 34.28 | 17.14 | 4.630 | 0.0910 |
| 00Midstory | Ligastrum | - | - | - | - | - |
| Midstory | Ostrya | 1 | 28.71 | 28.70 | 8.140 | **0.01210** |
| Midstory | Quercus | - | - | - | - | - |
| Midstory | Tilia | - | - | - | - | - |
| Midstory | Ulmus | 1 | 0.6670 | 0.6670 | 0.1340 | 0.7330 |
| Canopy | Q_alba | 2 | 340.0 | 169.8 | 2.955 | 0.05970 |
| Canopy | Q_rubra | 2 | 37.60 | 18.79 | 0.3930 | 0.6790 |

Table S2. Soil properties (mean ± 1 SE) across the restoration chronosequence. Variables in italic denote those included in the principal component analysis. Letters after means denote significant differences among sites.

| Variable | Young | Intermediate | Old | F | P |
| --- | --- | --- | --- | --- | --- |
| *pH* | 5.234±0.204 | 5.225±0.202 | 5.252±0.206 | 0.788 | 0.484 |
| NA meg/100g | 4.125±0.800 | 3.875±0.473 | 3.000±0.540 | 0.905 | 0.438 |
| *OM (%)* | 2.875±0.095 | 3.150±0.444 | 2.975±0.38 | 0.166 | 0.850 |
| *Bray-P1/Kg Ha* | 16.00±1.47 **(a)** | 30.00±2.121 **(ab)** | 20.00±3.08 **(b)** | 9.65 | **0.00600** |
| *Ca/Kg Ac* | 1132±125 | 1142±263 | 1156±460 | 0.616 | 0.562 |
| *Mg/Kg Ac* | 537.5±82.7 | 409.8±74.2 | 525.3±94.7 | 0.699 | 0.520 |
| *K/Kg Ac* | 132.3±7.33 **(a**) | 210.5±9.44 **(b**) | 171.5±9.42 **(b)** | 19.8 | **0.000500** |
| *CEC meq/100g* | 9.375±0.437 | 8.725±1.03 | 9.325±1.24 | 0.141 | 0.870 |
| Total C (%) | 1.825±0.0560 | 2.063±0.383 | 3.580±1.12 | 1.91 | 0.198 |
| Total N (%) | 0.163±0.00600 | 0.185±0.0328 | 0.2940±0.0884 | 1.65 | 0.241 |
| C/N ratio | 11.23±0.00700 | 10.99±0.00200 | 12.11±0.00500 | 2.26 | 0.154 |
| Sand (%) | 15.00±1.77 | 11.88±1.19 | 15.63±1.19 | 2.02 | 0.188 |
| *Silt (%)* | 75.63±2.95 | 80.63±1.19 | 75.00±1.77 | 2.15 | 0.173 |
| Clay (%) | 9.375±0.625 | 9.000±0.000 | 9.375±1.19 | 1.93 | 0.201 |

Table S3. Primers and target regions used to identify ASVs and microbial taxa from soil

|  | **515F–806R** | **ITS1f-ITS2** | **AMV4.5NF (F)/AMDGR (R)** |
| --- | --- | --- | --- |
| **Taxa** | **Bacteria** | **Fungi** | **Fungi (AMF enhanced)** |
| **Target region** | V4 region of 16S | ITS1f/ITS2 region of nuclear ribosomal RNA genes | SSU 18S rRNA gene |
| **Forward** | GTGYCAGCMGCCGCGGTAA | TTGGTCATTTAGAGGAAGTAA | AAGCTCGTAGTTGAATTTCG |
| **Reverse** | GGACTACNVGGGTWTCTAAT | GCTGCGTTCTTCATCGATGC | CCCAACTATCCCTATTAATCAT |

Table S4. Microbial community characteristics of conditioned soil across plant species. We report Shannon diversity of both fungal and bacterial taxa, and the results of the indicator analyses using IndVal, which includes the total number of unique core microbial taxa for plants grown in soil inocula.

| **Plant Species** | **Soil Type** | **# Unique microbial taxa** |
| --- | --- | --- |
| GV | Young | 15 |
| GV | Intermediate | 44 |
| GV | Old | 6 |
| SA | Young | 2 |
| SA | Intermediate | 14 |
| SA | Old | 62 |
| SC | Young | 44 |
| SC | Intermediate | 1 |
| SC | Old | 4 |

Table S5. Results of the SIMPER analysis of fungal and bacterial phyla within and between species conditioned in three soil inocula. *Contrib* denotes the percent a particular phyla contributes to overall Bray Curtis dissimilarity between communities, while *Abund* denotes the species x soil combination that is driving this contribution to beta diversity.

| ***G. virginianum vs S. arguta*** | | | | | | | | |
| --- | --- | --- | --- | --- | --- | --- | --- | --- |
| **Young** | | | **Intermediate** | | | **Old** | | |
| **Phyla** | **Contrib** | **Abund** | **Phyla** | **Contrib** | **Abund** | **Phyla** | **Contrib** | **Abund** |
| Cyanobacteria | 5.79% | SA | Firmicutes | 29.11% | GV | Firmicutes | 29.36% | Varies |
| Firmicutes | 33.50% | SA | Proteobacteria | 1.556% | GV | Cyanobacteria | 1.611% | SA |
| Proteobacteria | 3.93% | SA | Myxococcota | 1.233% | SA | Myxococcota | 1.384% | SA |
| Ascomycota | 53.17% | Varies | Ascomycota | 55.79% | Varies | Ascomycota | 65.83% | Varies |
| Glomeromycota | 4.49% | GV | Glomeromycota | 2.906% | GV | Chytridiomycota | 5.056% | GV |
| Unidentified | 1.67% | GV |  | | | Unidentified | 3.610% | Varies |
|  | | |  |  |  | Rozellomycota | 2.121% | SA |
| ***G. virginianum* vs *S. caesia*** | | | | | | | | |
| **Young** | | | **Intermediate** | | | **Old** | | |
| **Phyla** | **Contrib** | **Abund** | **Phyla** | **Contrib** | **Abund** | **Phyla** | **Contrib** | **Abund** |
| Firmicutes | 5.54% | GV | Myxococcota | 2.062% | GV | Firmicutes | 13.97% | SC |
| Cyanobacteria | 2.70% | SC | Proteobacteria | 4.987% | GV | Proteobacteria | 2.468% | SC |
| Chloroflexi | 1.35% | SC | Firmicutes | 11.64% | SC | Acidobacteriota | 1.819% | SC |
| Ascomycota | 38.44 | Varies | Gemmatimonadota | 1.001% | GV | Gemmatimonadota | 1.277% | SC |
| Basidiomycota | 13.77% | SC | Acidobacteria | 1.202% | GV | Ascomycota | 56.07% | Varies |
| Glomeromycota | 11.56% | SC | Ascomycota | 64.38% | Varies | Basidiomycota | 15.27% | SC |
|  | | | Basidiomycota | 10.78% | SC | Chytridiomycota | 4.178% | GV |
|  |  |  | Glomeromycota | 2.928% | GV | Unidentified | 1.412% | GV |
| ***S. arguta vs S. caesia*** | | | | | | | | |
| **Young** | | | **Intermediate** | | | **Old** | | |
| **Phyla** | **Contrib** | **Abund** | **Phyla** | **Contrib** | **Abund** | **Phyla** | **Contrib** | **Abund** |
| Firmicutes | 32.27% | SA | Firmicutes | 40.58% | Varies | Firmicutes | 26.95% | SA |
| Cyanobacteria | 6.09% | SA | Proteobacteria | 1.058% | SA | Cyanobacteria | 1.920% | SA |
| Proteobacteria | 4.23% | SA | Ascomycota | 46.40% | Varies | Myxococcota | 1.612% | SA |
| Ascomycota | 38.44% | Varies | Basidiomycota | 13.10% | SC | Ascomycota | 56.92% | Varies |
| Basidiomycota | 13.71% | SC |  | | | Basidiomycota | 19.92% | SC |
| Glomeromycota | 11.56% | Varies |  |  |  | Rozellomycota | 2.004% | SA |
| Unidentified | 6.95% | SC |  |  |  | Glomeromycota | 1.027% | SC |


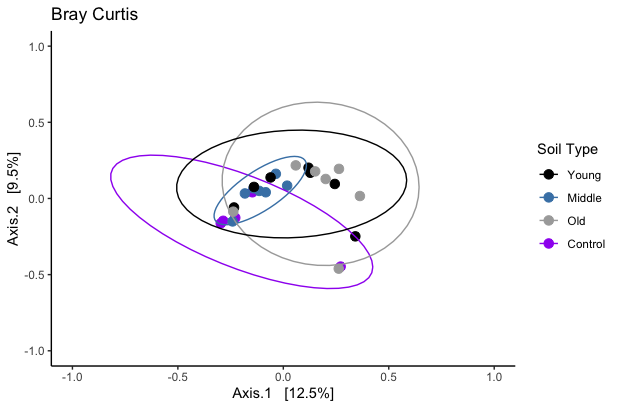

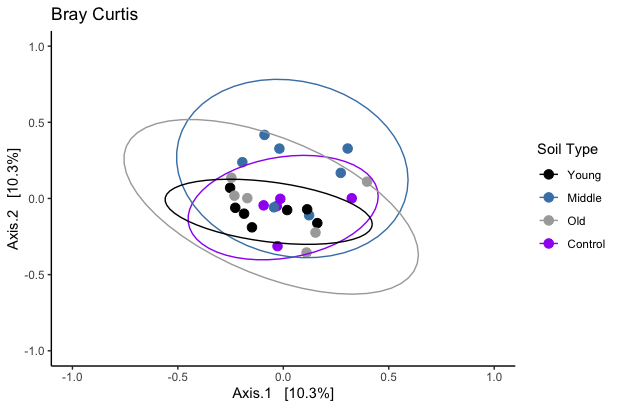

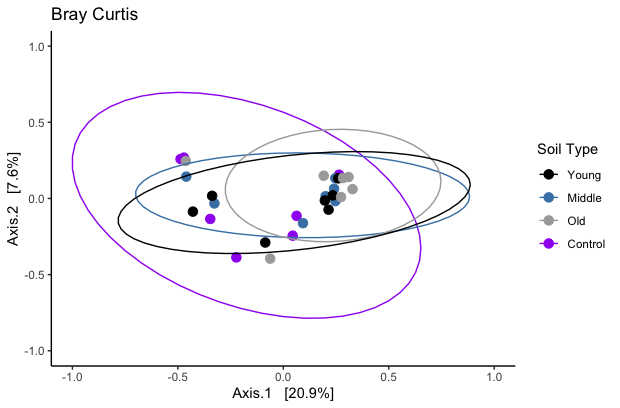

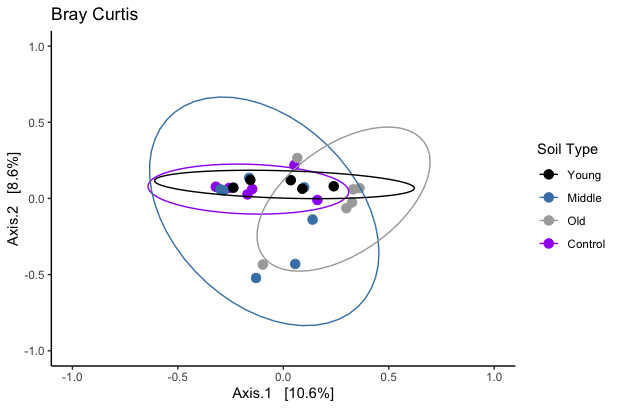

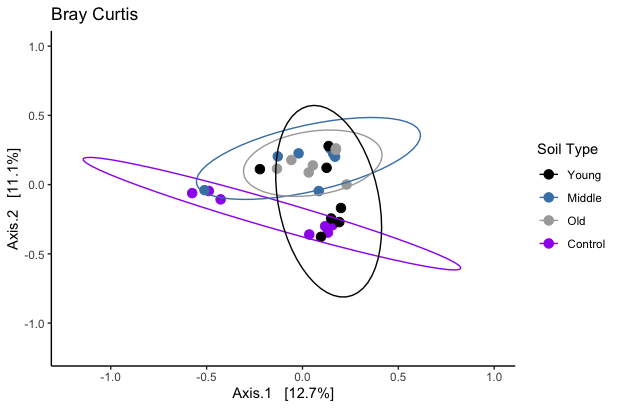

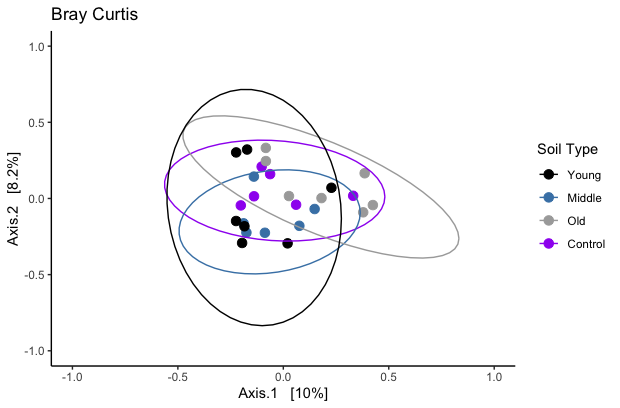


SA Fungi

GV Bacteria

SA Bacteria

GV Fungi

SC Bacteria

SC Fungi

Figure S1. NMDS ordination plots based upon Bray- Curtis dissimilarity of microbial community composition for three native plant species grown in each soil inocula.


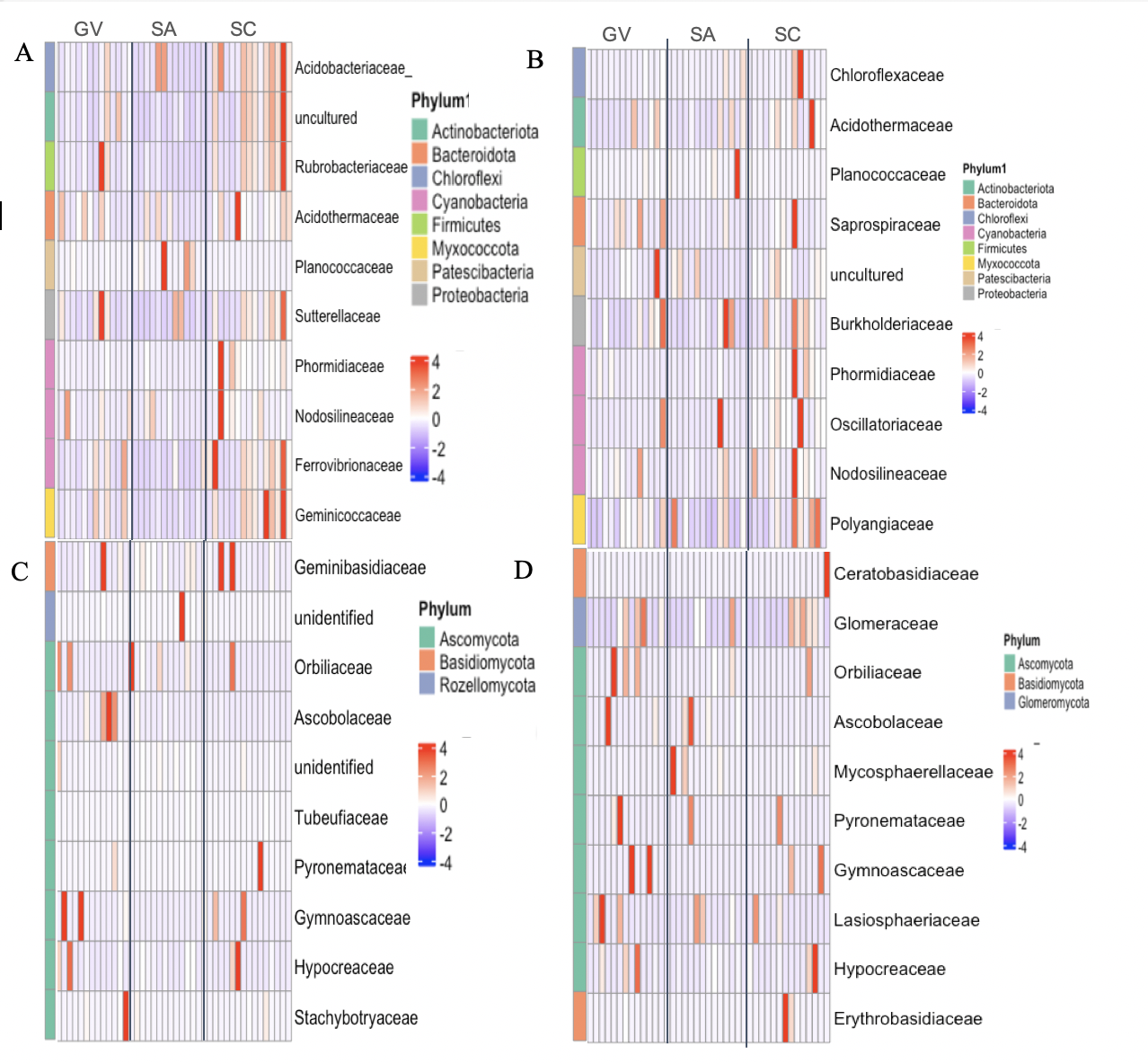


Figure S2. Heatmaps of top ten significantly differentially abundant taxa across soils conditioned by three focal plant species (GV, SA, SC) sorted by phyla and family. 5a) depicts bacterial taxa from conditioned soil from plants grown in young compared to old soil, 5b) depicts bacterial taxa of conditioned soil from plants grown in young compared to intermediate soil, 5c) depicts fungal taxa of conditioned soil from plants grown in young compared to old soil, and 5d) depicts fungal taxa of conditioned soil from plants grown in young compared to intermediate soil. Red indicates when a taxon group is significantly abundant in young soil compared to those grown in old or intermediate soil.
